# Late-onset bullous keratopathy after iris-fixated phakic intraocular lens implantation: a case report

**DOI:** 10.1186/s12886-026-04899-2

**Published:** 2026-06-02

**Authors:** Salmaan Ferrand, Anna Bogdanova-Bennett, Daniel T. Cornelius, Rashida Abbas Ferrand

**Affiliations:** 1 Sunderland Royal Hospital, Kayll Road, Sunderland, SR4 7TP UK; 2 Newcastle Eye Centre, Royal Victoria Infirmary, Newcastle-Upon-Tyne, Tyne and Wear, NE1 4LP UK; 3 Pretoria Eye Institute, Pretoria, South Africa; 4 Clinical Research Department, London School of Hygiene and Tropical Medicine, Keppel Street, London, WC1E 7HT UK

**Keywords:** Anterior chamber phakic intraocular lens, Endothelial decompensation, Bullous keratopathy, Descemet membrane endothelial keratoplasty

## Abstract

**Background:**

This article presents a case report of bullous keratopathy occurring 12 years after implantation of iris-fixated anterior chamber phakic intraocular lenses (AC pIOL), which eventually required Descemet Membrane Endothelial Keratoplasty (DMEK). This represents a much longer period of endothelial decompensation following implantation than that reported in other studies.

**Case presentation:**

A 44-year-old woman presented with pain and reduced vision which was diagnosed as uveitis; it was unresponsive to topical steroids. Twelve years earlier, she had iris-fixated AC pIOLs (Myopia Artiflex 6 mm) implanted in both eyes for correction of myopia. Substantial endothelial loss with a bullous keratopathy was identified and both intraocular lenses were explanted. Owing to endothelial decompensation in the left eye, the patient required a left Descemet Membrane Endothelial Keratoplasty.

**Conclusions:**

As the same models of AC pIOLs are still in use today, clinicians should be aware of this serious complication when considering different refractive error correction procedures. Our report highlights that endothelial decompensation as a complication of AC pIOL implantation can occur many years after lens implantation, and emphasizes the need for long-term endothelial monitoring.

## Background

Anterior chamber phakic intraocular lenses (AC pIOLs) are intraocular lenses that are either enclavated onto midperipheral immobile regions of the iris (“iris-fixated”) or stabilized by their haptics within the irido-corneal angle (“angle-supported”) to correct ametropia while retaining the patient’s natural lenses [1]. pIOLs do not compromise accommodation like refractive lens exchanges, are theoretically reversible through explantation, and offer equivalent, if not superior, refractive correction in situations where laser refractive surgery is contraindicated such as in patients with reduced corneal thickness [1, 2].

However, they are also associated with adverse effects such as intraocular pressure elevation and corneal endothelial cell loss [2–7]. Implantable Collamer Lenses (ICLs) are alternative phakic intraocular lenses that are inserted within the posterior chamber, behind the iris but in front of the lens. Their distance from the endothelium reduces the risk of endothelial decompensation and are considered safer than refractive lens exchanges (RLEs) where the crystalline lens is replaced with a custom lens of a different refractive power. This is associated with retinal detachment and loss of accommodation [1, 2].

We report a case of bullous keratopathy that required a keratoplasty more than a decade after bilateral AC pIOL implantation.

## Case presentation

In December 2006, a 32-year-old woman presented to an eye clinic with a refraction of -7.25 / -0.25 × 10 in the right eye and − 8.00 / -0.50 × 170 in the left requesting laser treatment with an aim to be completely free of glasses for distance correction. Prior to surgery, the patient had an endothelial cell density (ECD) of 2770 cells/mm^2^ in the left eye and 3167 cells/mm^2^ in the right eye on specular microscopy. Their anterior chamber depth (ACD) was 3.60 mm and 3.61 mm, which corrected to 2.80 mm and 2.81 mm after subtracting corneal thickness, in their left and right eyes respectively. A decision was made for AC pIOL implantation because the patient’s thin corneas made them unsuitable for LASEK. Two iris-fixated AC pIOLs (6 mm Myopia Artiflex) were implanted into both eyes and, after 5 months, the patient had bilateral plano vision. The patient was monitored for several months but was subsequently lost to follow-up.

**Fig. 1:**
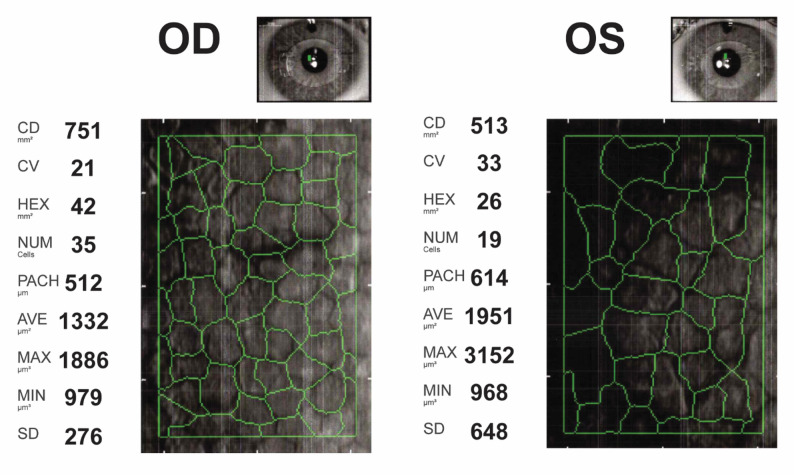
Specular microscopy with auto-tracing of corneal endothelial cells in the right (*left*) and left (*right*) eye showing signs of polymegethism and pleomorphism in a patient presenting 12 years after anterior chamber phakic intraocular lens implantation

Twelve years later, in 2019, the patient presented with painful red eyes and blurred vision and was diagnosed with uveitis which was managed with topical steroids. Their symptoms resolved partially; they presented to an eye hospital a few months later in October 2019 with persistent discomfort in the left eye. They had a visual acuity of 6/6 and refraction of -1.00 / -0.75 × 120 in the left eye and − 0.75 / -0.75 × 88 in the right eye. On pachymetry, their minimal minimum corneal thickness was 517 μm with an ACD (including corneal thickness) of 3.22 mm in the right eye and 582 μm with an ACD of 3.31 mm in the left eye. Specular microscopy showed an endothelial cell count of 513 cells/mm^2^ in the left eye and 751 cells/mm^2^ in the right with signs of polymegethism and pleomorphism (Fig. 1).

Given the degree of corneal endothelial cell loss, the patient was listed for pIOL explantation in early November 2019 and received topical Predforte twice daily with saline eye drops 2-hourly in the interim. The left pIOL was first explanted followed by the right AC pIOL three days later. In both operations, the haptics of the IOLs were manipulated with a Sinsky hook through the main incision and folded out well. The explantation procedures and follow-up periods were both uneventful. The patient’s subsequent refraction was − 7.75 / -0.50 × 170 in the right eye and − 10.00 / -1.00 × 150 in the left, and glasses were prescribed for correction of vision. The visual acuity was 6/48 in the right eye and 6/30 in the left.

**Fig. 2:**
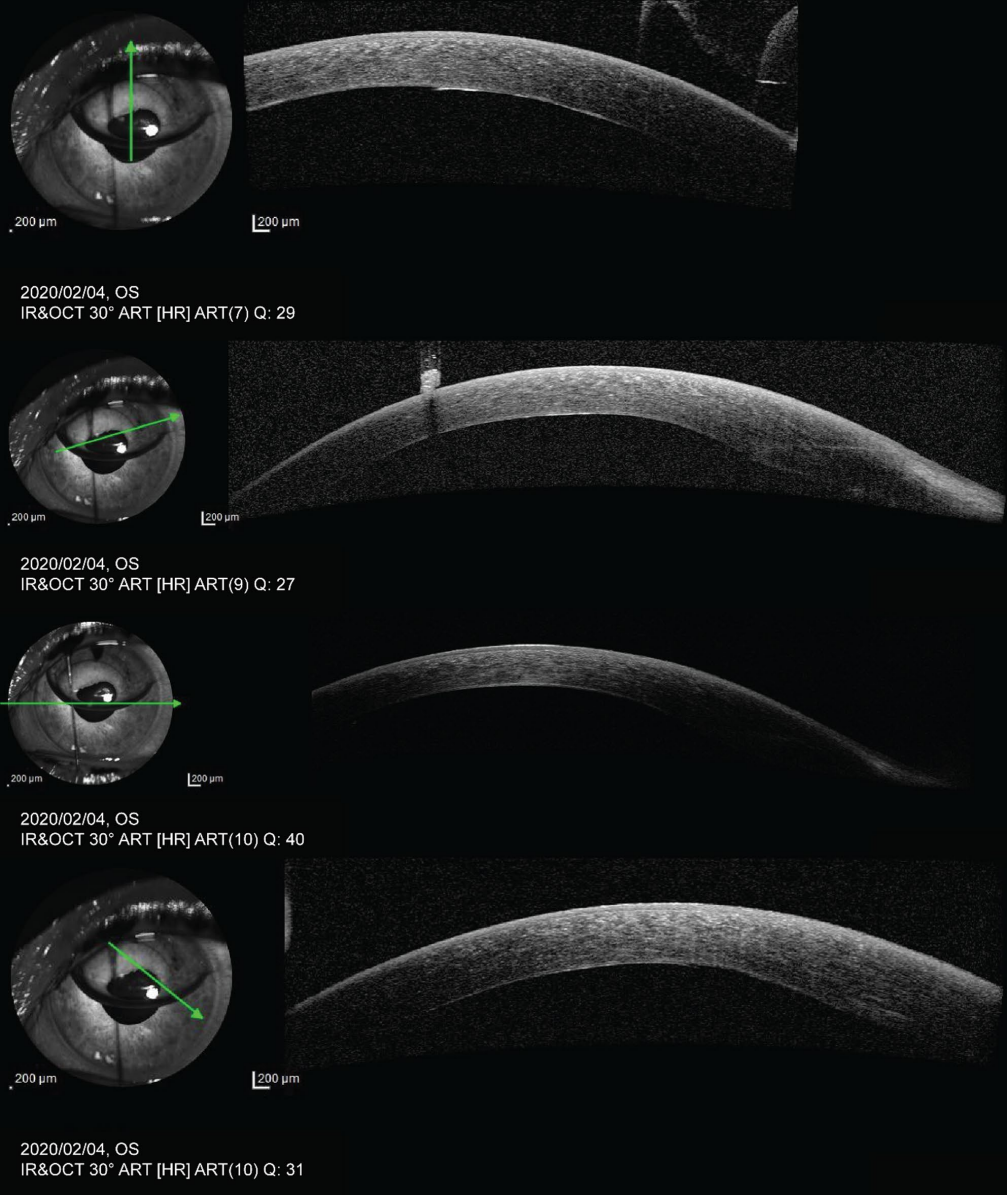
Anterior segment optical coherence tomography of the patient’s left eye 1 day after a left Descemet Membrane Endothelial Keratoplasty. Central Corneal thickness measures 510 μm

The patient was scheduled for a follow-up in 3 months but returned a month earlier in January 2020 complaining of acute visual loss in the left eye. The left eye visual acuity was 6/60 and, on examination, there were signs of endothelial decompensation with bullous keratopathy due to the reduced ECD. The patient was scheduled for a left Descemet Membrane Endothelial Keratoplasty (DMEK) 2 weeks later in early February 2020. The result was captured 1 day post-operatively by anterior segment Optical Coherence Tomography (Fig. 2). A central corneal thickness of 510 μm was measured.

**Fig. 3:**
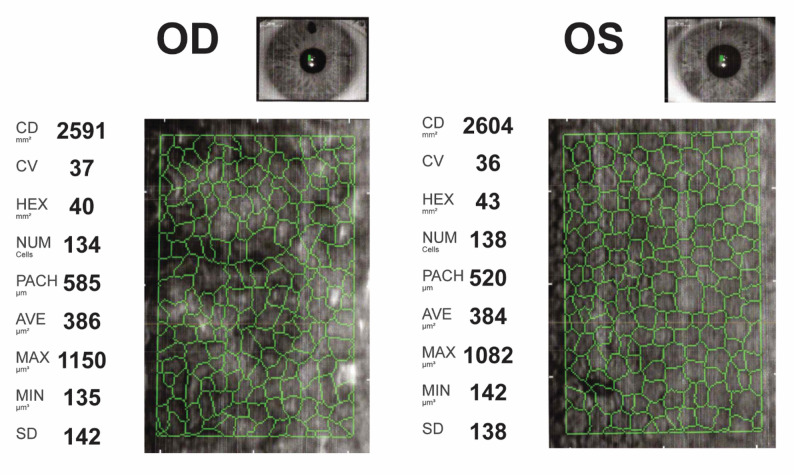
Specular microscopy with auto-tracing of corneal endothelial cells in the right (*left*) and left (*right*) eye after bilateral explantation of anterior chamber iris-fixated phakic intraocular lenses and a Descemet Membrane Endothelial Keratoplasty in the left eye

After surgery, the patient’s visual acuity in the left eye corrected to 6/7.5. At follow-up nine month later, refraction was − 8.25/-0.50 × 90 in the right eye and − 9.00/-1.25 × 51 on the left with uncorrected visual acuity of 6/60 in both eyes. Pachymetry showed that the minimum corneal thickness was 558 μm with an ACD of 3.38 mm in the right eye and 504 μm with an ACD of 3.40 mm in the left eye. Specular microscopy showed that the patient had made a recovery with an ECD of 2410 cells/mm^2^ in the left eye and 2591 cells/mm^2^ in the right eye (Fig. 3).

## Discussion

This report highlights bullous keratopathy as a rare but serious complication of AC pIOL implantation. While endothelial cell loss has been documented in various case reports [8, 9], to our knowledge there has been only one other report of endothelial cell loss resulting in bullous keratopathy severe enough to require a DMEK more than a decade after implantation of AC pIOLs [10].

Reduction of ECD is a recognized complication of AC pIOLs [1]. Potential reasons include acute loss through intra-operative surgical damage [3–5], or chronically through background physiological loss [11], subclinical chronic inflammation, changes to aqueous flow [6], or direct damage of endothelial cells from contact between the AC pIOL and the corneal endothelium. It has been proposed that intermittent contact may occur during eye rubbing or sleep when their closed eyes press against a pillow [5]. Contact is seldom constant and may not necessarily be identified on examination or anterior chamber optical coherence tomography.

A shallow anterior chamber may elevate the risk of AC pIOLs irritating the corneal endothelium [3, 4, 7]. Consequently, studies recommend against implanting AC pIOLs in patients with a low ACD (defined as the distance between the corneal epithelium and the anterior crystalline lens capsule) with variable ACD thresholds for eligibility across studies e.g. 2.8 mm [3, 7], 3mm [11], or 3.2mm [5]. In this case, the patient’s corrected ACD, which measured 2.80 mm and 2.81 mm in the left and right eyes respectively, could be considered a contraindication to AC pIOL implantation.

Following endothelial cell loss in the corneal peripheries, there is compensatory migratory movement of normal endothelial cells. Remaining cells enlarge and overall ECD decreases. This compromises endothelial pump action, resulting in stromal oedema which eventually form blisters or bullae [9]. AC pIOLs can, however, increase the normal death rate of endothelial cells without creating a bullous keratopathy.

In this patient there was an 81.5% and 76.3% loss of endothelial cell density in their left and right eyes respectively, with bullous keratopathy and subsequent visual loss occurring 12 years after original lens implantation. The only ocular history of note in that time was an episode of uveitis that responded to steroid therapy. This may have been idiopathic, heralded the development of corneal endothelial decompensation, or itself have contributed to endothelial cell loss. When the patient was seen in October 2019 however, there were no clinical signs of anterior uveitis. The patient’s initial episode of anterior uveitis may therefore have been a misdiagnosis of pseudophakic bullous keratopathy.

The American Academy of Ophthalmology published a special report with guidelines regarding pre-operative and post-operative ECD measurements. Pre-operatively, AC pIOLs are recommended if ECD > 2500 cells/mm^2^ in those older than 21 years old or > 2000 cells/mm^2^ if older than 40 years old [1]. Post-operatively, any loss over 20% or an ECD < 1500 cells/mm^2^ should prompt further investigation [12].

Once the pIOLs were explanted, the ECD improved in the right eye, but the left eye required a DMEK with subsequent recovery, demonstrating the suitability of DMEK as a therapeutic strategy for bullous keratopathy occurring as a sequelae of iris-fixated AC pIOL implantation. The left eye likely lost endothelial cells beyond a critical threshold, depleting the reserve capacity necessary for cellular redistribution and corneal healing. Conversely, the right eye’s low initial count may have reflected the measurement of a localized island of reduced cell density, allowing time for recovery through migration of neighboring healthy endothelial cells.

While some studies stress that they have not found significant ECD loss and have thus advocated for the long-term safety and efficacy of iris-fixated AC pIOLs [11], this case highlights a serious, albeit rare, vision-threatening complication, of which clinicians need to be aware.

Clinicians should counsel patients about the risks and benefits of AC pIOL implantation, including detailed discussion about alternative available procedures to correct refractive error, such as ICLs or RLEs. ICLs are associated with a lower risk of endothelial cell loss. For this patient, the calculus of choosing an AC pIOL over an ICL may have been due to surgeon preference, experience, or a lack of awareness of the risks to the endothelium in 2006. Nevertheless, patients who still prefer to use AC pIOLs should receive life-long follow-up with annual specular microscopy and ECD monitoring.

## Data Availability

Data sharing is not applicable to this article as no datasets were generated or analysed during the current study.

## Declarations

### Value statement

What Was Known - Corneal Decompensation is a recognized complication of anterior chamber phakic intraocular lens implantation. To the Authors’ knowledge, there has only been one other case report of endothelial decompensation requiring an AC pIOL explantation and a subsequent DMEK more than a decade after AC pIOL implantation. What This Paper Adds - This paper reports the development of bullous keratopathy requiring a keratoplasty as a complication of anterior chamber phakic intraocular lens implantation, occurring more than a decade after the original refractive correction procedure, which is a longer onset than what has been reported in other studies and case reports. The long timeframe within which this complication can occur emphasizes the need for careful patient counselling and regular lifelong monitoring with annual specular microscopy post-operatively. This report also provides insight into the management of severe bullous keratopathy in a patient with iris-fixated anterior chamber phakic intraocular lenses.

## Abbreviations

ACD: Anterior chamber depth
AC pIOL: Anterior chamber phakic intraocular lens
DMEK: Descemet membrane endothelial keratoplasty
ECD: Endothelial cell density
ICL: Implantable collamer lens
RLE: Refractive lens exchange
